# Comparing Magnetic Resonance Fingerprinting (MRF) and the MAGiC Sequence for Simultaneous T1 and T2 Quantitative Measurements in the Female Pelvis: A Prospective Study

**DOI:** 10.3390/diagnostics13132147

**Published:** 2023-06-23

**Authors:** Bo-Syuan Huang, Ching-Yi Hsieh, Wen-Yen Chai, Yenpo Lin, Yen-Ling Huang, Kuan-Ying Lu, Hsin-Ju Chiang, Rolf F. Schulte, Chien-Yuan Eddy Lin, Gigin Lin

**Affiliations:** 1 Department of Medical Imaging and Intervention, Chang Gung Memorial Hospital at Linkou, Taoyuan 33382, Taiwan; 2 Medical Imaging Research Center, Institute for Radiological Research, Chang Gung University, No.259, Wenhua 1st Rd., Guishan Dist., Taoyuan City 33302, Taiwan; 3 Department of Medical Imaging and Radiological Sciences, Chang Gung University, 5 Fuhsing St., Guishan, Taoyuan 33382, Taiwan; 4 Clinical Metabolomics Core Laboratory, Chang Gung Memorial Hospital at Linkou, 5 Fuhsing St., Guishan, Taoyuan 33382, Taiwan; 5 GE Healthcare, 80807 Munich, Germany; 6 GE Healthcare, Taipei 10480, Taiwan

**Keywords:** computer-assisted image processing, fingerprinting, gynecology, magnetic resonance imaging

## Abstract

The aim of this study was to explore the potential of magnetic resonance fingerprinting (MRF), an emerging quantitative MRI technique, in measuring relaxation values of female pelvic tissues compared to the conventional magnetic resonance image compilation (MAGiC) sequence. The study included 32 female patients who underwent routine pelvic MRI exams using anterior and posterior array coils on a 3T clinical scanner. Our findings demonstrated significant correlations between MRF and MAGiC measured T1 and T2 values (*p* < 0.0001) for various pelvic tissues, including ilium, femoral head, gluteus, obturator, iliopsoas, erector spinae, uterus, cervix, and cutaneous fat. The tissue contrasts generated from conventional MRI and synthetic MRF also showed agreement in bone, muscle, and uterus for both T1-weighted and T2-weighted images. This study highlights the strengths of MRF in providing simultaneous T1 and T2 mapping. MRF offers distinct tissue contrast and has the potential for accurate diagnosis of female pelvic diseases, including tumors, fibroids, endometriosis, and pelvic inflammatory disease. Additionally, MRF shows promise in monitoring disease progression or treatment response. Overall, the study demonstrates the potential of MRF in the field of female pelvic organ imaging and suggests that it could be a valuable addition to the clinical practice of pelvic MRI exams. Further research is needed to establish the clinical utility of MRF and to develop standardized protocols for its implementation in clinical practice.

## 1. Introduction

Medical imaging plays a crucial role in precision medicine by providing quantifiable features [1]. Magnetic resonance imaging (MRI) is widely used for female pelvic imaging due to its excellent soft tissue contrast and multiplanar imaging capability [2]. However, conventional quantitative MRI is time-consuming, and imaging registration can be affected by interscan motion. Magnetic resonance fingerprinting (MRF) is a promising approach that generates quantitative tissue property maps in a single, efficient acquisition by using variable flip angles and repetition times [3]. Although still in development, MRF has demonstrated potential in simultaneously measuring multiple parameters, such as T1, T2, relative spin density, and B0 inhomogeneity in various organs, including the brain [4,5,6], liver [7,8], kidney [9], prostate [10,11], breast [12] and heart [13]. MRF has been effective in differentiating common types of adult intra-axial brain tumors [4], exploring new patterns of the neuroradiology [5], and classifying meningiomas into soft, moderate, and hard ones for further surgical planning [6]. MRF also allows quantitative measurement of liver and kidney parenchyma in a single breath-hold scan [7], with the good agreement and correlation with conventional mapping methods [8]. By combining MRF-derived values, we can detect prostate cancer and identify the difference between high- and low-grade cancers [10]. Significant associations between MRF quantitative parameters and tissue compartments on prostate cancer, prostatitis, and normal peripheral zones have also been observed [11]. In breast images, MRF measurement of parameters is repeatable, reproducible, and may play a role in the clinical application [12]. Moreover, myocardial T1 and T2 mapping generated from MRF is comparable to conventional mapping sequence [13]. However, there has been no systematic evaluation of MRF in female pelvic structures.

Magnetic Resonance Image Compilation (MAGiC), on the other hand, is a technique that allows for the acquisition of multiple images contrasts in a single MRI scan by acquiring several images with varying contrast weightings, including T1-weighted, T2-weighted, and proton density-weighted images. This approach is primarily used in neuroimaging [14] but has been extended to other anatomical regions, such as the breast [15], spine [16], and rectum [17]. In breast imaging, a positive correlation between T2 relaxation time by MAGiC and by multi-echo spin-echo mapping method has been reported in breast cancer patients [15]. Another study compared the diagnostic image quality of synthetic images generated by MAGiC to conventional imaging in the lumbar spine and concluded that MAGiC provided more quantitative information while maintaining comparable image quality [16]. A recent study investigated the use of MAGiC in locally advanced rectal cancer patients who had undergone neoadjuvant chemoradiotherapy. The study found that T1 and T2 values generated by MAGiC were lower in patients with a complete response and T-downstage [17]. However, no studies have reported T1 and T2 values for structures in the female pelvis, making the current study a unique contribution to the field.

The objective of this prospective study was to examine the relaxation values of the female pelvis using MRF and compare the obtained quantitative T1 and T2 values with those obtained from the MAGiC sequence.

## 2. Materials and Methods

### 2.1. Participant Patients

**Figure 1:**
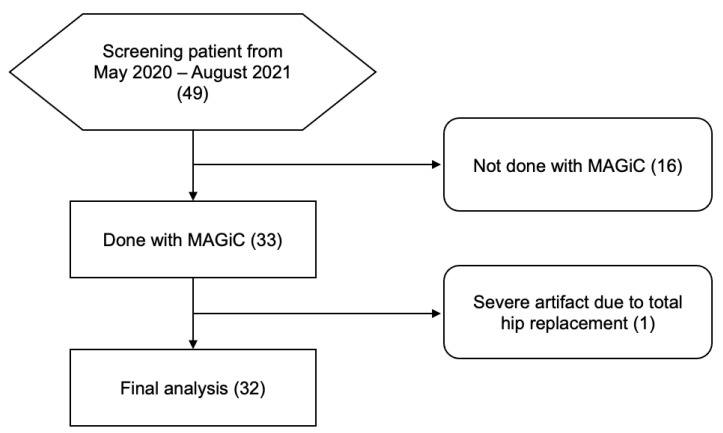
Flow diagram of patient selection. Parentheses indicate the case number. MAGiC, magnetic resonance image compilation.

We prospectively enrolled 49 female patients between May 2020 and August 2021 who were scheduled for routine pelvic MRI exams. Informed consent was obtained from all study subjects, and the study was approved by the Chang Gung Institutional Review Board (IRB 201702080A0C601). The inclusion criteria were females aged over 20 years who were either in pre-operative or post-operative status for gynecological disease. The exclusion criteria included patients without available MRF or available MAGiC and suboptimal MR image quality. The study flow diagram is shown in Figure 1.

### 2.2. MRI Methods

A 3T clinical scanner (MR 750W, GE Healthcare, Waukesha, WI, USA) with anterior and posterior array coils was used to perform all MRI acquisitions. A 2D fast-spin-echo multi-saturation-delay multi-echo is employed for MAGiC acquisition. MRF was acquired using Steady-state free precession (SSFP), and the acquisition trajectories utilized under-sampled golden-angle spiral interleaves with sampling bandwidth = ±250 kHz, TE = 2.2 ms, NEX = 1, and 979 frames. The variable repetition time and scan flip angle list were adapted from Jiang et al. [18]. A slice-selective inversion pulse was used before the flip angle and TR list variation. The dictionary did not contain the static magnetic field (B0) or the transmit radiofrequency field (B1+). To improve T2 accuracy, a slice profile was included. Other imaging parameters were controlled identically in both MAGiC and MRF scans: FOV = 22 mm × 22 mm; matrix = 256 × 256; slice thickness = 4 mm with 1-mm gap; 20 slices. The scan time for MRF and MAGiC were 3.6 min and 4 min, respectively.

Conventional MRI was also performed in the same study session. In brief, T1-weighted and T2-weighted turbo spin-echo sequences were applied with specific parameters: for T1, a repetition time (TR) of 626 ms and an echo time (TE) of 11 ms, with 2 signal averages and a matrix of 256 × 320, and a field of view (FOV) of 20 cm; for T2, a TR of 5630 ms and a TE of 87 ms, with 3 signal averages and a matrix of 256 × 320, and a FOV of 20 cm. Diffusion-weighted imaging (DWI) was carried out using a single-shot echo-planar technique with fat suppression, with a TR of 3300 ms and a TE of 79 ms, 4 signal averages, a matrix of 128 × 128, and a FOV of 20 cm, in 5-mm trans-axial plane. The diffusion-weighted gradients were applied orthogonally in slice-selective, phase encoding, and read-out directions. Apparent diffusion coefficient (ADC) maps were generated from isotropic diffusion-weighted images with b values of 0 and 1000 s/mm^2^. Contrast-enhanced T1WI was acquired after intravenous injection of 0.1 mmol/kg body weight of contrast medium (Gadopentetate dimeglumine, Magnevist, Schering, Berlin, Germany) followed by a 20-mL saline flush, at about 120–180 s equilibrial phases. The T1WI was acquired with a TR of 567 ms, a TE of 10 ms, a flip angle of 150°, 2 signal averages, a matrix of 256 × 320, and a FOV of 20 cm. The scan was performed during normal respiration and minimal breathing, and no premedication was administered.

### 2.3. Image Analysis

**Figure 2:**
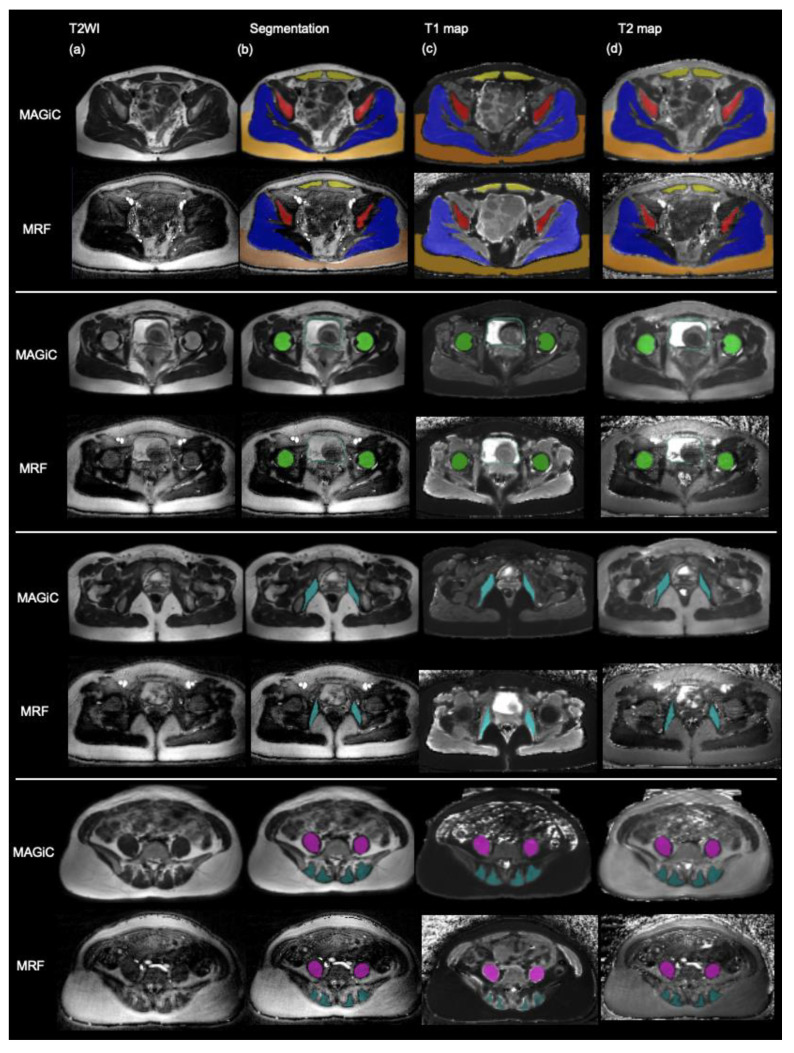
Schematic diagram of drawing ROIs. (**a**) Synthetic axial T2WI from MAGiC (upper row) and MRF (lower row). (**b**) Segmentation: Drawing ROIs on synthetic axial T2WI with the full extent of structures. (**c**) Pasting the segmentations to the corresponding T1 map. (**d**) Pasting the segmentations to the corresponding T2 map. MAGiC, magnetic resonance image compilation; MRF, Magnetic resonance fingerprinting.

Quantitative MAGiC T1 and T2 maps were generated from the MAGiC raw image dataset using SyMRI 8 software (SyntheticMR AB, Linköping, Sweden). MRF T1 and T2 maps were obtained by pattern matching of the T1 and T2 simulation dictionary with reconstructed time frames of the acquired data. The MRF dictionary was computed for T1 and T2 using the extended phase graphs formalism [18] and included the slice profile [19]. Regions of interest (ROIs) of tumor contours were delineated by the consensus of two radiologists (B-S.H and G.L., with 2 and 20 years of experience, respectively), using an open-source software ITK-SNAP (www.itksnap.org, assessed on 2 February 2022) [20]. The contouring was carefully made in detailed pelvic structures, including ilium, femoral head, gluteus, rectus abdominis, obturator, iliopsoas, erector spinae, uterus, cervix, bladder wall, cutaneous fat, for both MAGiC and MRF images. ROIs were taken by drawing in the synthetic axial T2-weighted imaging on multi-slice and then registered onto the corresponding T1 and T2 maps for quantitative analysis. A representative case is presented in Figure 2, which includes the femoral head, ilium, gluteus, rectus abdominis, obturator internus, psoas muscle, urinary bladder wall and cutaneous fat. ROI of other structures was also delineated in a single axial plane as possible. Besides, we only included cutaneous fat at and behind the gluteus major to discard any noise arising from artifacts at the anterior abdominal wall away from the body coil. Moreover, ROIs of the uterus and cervix were not obtained in post-operative patients, and for those patients with leiomyomas, leiomyomas were excluded when drawing the ROI of the uterus. Mean T1 and T2 values of all selected pelvic tissue were computed over all voxels [21].

In this study, different types of tissues were selected for a qualitative comparison between MRF and T1WI and T2WI. The categories of tissues included bone, muscle, fat, uterus, vessel, and urine. To compare the signal intensity characteristics of these tissues, the signal intensity of all tissues on T1WI and T2WI was defined in comparison to the fatty bone marrow in the pubic symphysis, which was considered as a reference [22]. The signal intensity pattern was divided into two categories, which were (1) iso- and hypo-intense and (2) hyperintense. To evaluate the agreement between MRF and T1WI, and T2WI, two radiologists with 11 and 8 years of experience in gynecological radiology independently interpreted the MR images. The agreement analysis was conducted by calculating Cohen’s kappa (κ) coefficient, which measures the inter-rater agreement beyond chance agreement. The κ coefficient ranges from 0 to 1, where 0 indicates poor agreement beyond chance and 1 indicates perfect agreement.

### 2.4. Statistical Analysis

For quantitative analysis, we calculated the Pearson correlation coefficient (r) to assess the correlation between T1 and T2 values obtained using MAGiC and MRF [23]. We also depicted a linear trend to visualize the correlation and calculated the significance of the correlation coefficient between the MRF and MAGiC measurements. To test for statistically significant differences between MRF-derived T1 or T2 values of tissues in patients with and without radiotherapy, the Mann–Whitney U test was performed.

For qualitative agreement analysis, we evaluated the agreement of tissue contrast between MRF and conventional T1WI and T2WI using weighted κ statistics. The statistics were used to classify the level of agreement between the two methods as poor agreement (0.00 < κ < 0.040), fair to the good agreement (0.40 ≤ κ ≤ 0.75), or excellent agreement (κ > 0.75) [24]. A corresponding *p*-value of less than 0.05 was considered statistically significant.

## 3. Results

### 3.1. Patient Cohort

The flow diagram in Figure 1 outlines the selection process for the study population. Initially, all patients who had undergone MRIs for evaluation of their hip joint were considered for inclusion in the study. However, 16 patients were excluded because they did not have an available axial plane in their MRI scan, and one patient was excluded due to a severe artifact caused by a total hip replacement. Ultimately, a total of 32 patients were included in the final analysis. The age of the participants ranged from 26 to 83 years, with a mean age of 49 years old. The study population consisted of patients in both pre-operative (n = 15) and post-operative (n = 17) status, reflecting a diverse patient population.

Table 1 presents the demographic and clinical characteristics of the study population. The data includes information on the patient’s age, gender, body mass index (BMI), and duration of hip pain. Additionally, the table provides details on the type of hip pathology observed in each patient, as well as the side of the affected hip. Overall, the study population is representative of a diverse group of patients with pelvic diseases, and the demographic and clinical characteristics provide valuable insights into the patient population studied.

### 3.2. Quantitative Comparison between MRF and MAGiC

**Figure 3:**
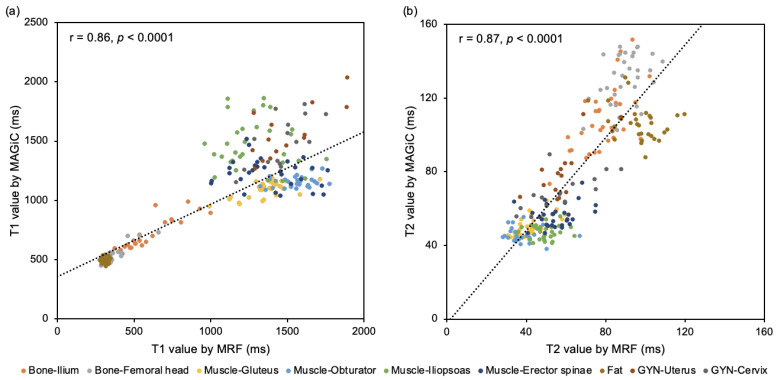
Correlation between MRF and MAGiC-derived T1 and T2 values. (**a**) Scatterplot with a correlation coefficient between MRF-derived T1 and MAGiC-derived T1; (**b**) Scatterplot with a correlation coefficient between MRF-derived T2 and MAGiC-derived T2. MRF, Magnetic resonance fingerprinting; MAGiC, magnetic resonance image compilation.

The study observed significant correlations between the MRF and MAGiC measured T1 and T2 values for various tissues, including the ilium, femoral head, gluteus, obturator, iliopsoas, erector spinae, uterus, cervix, and cutaneous fat. These correlations were considered statistically significant, implying that there is a strong relationship between the MRF and MAGiC-measured T1 and T2 values for these tissues. Table 2 provides the mean T1 and T2 values for all chosen tissues, categorized into bone, muscle, gynecological organ, bladder, and fat. These values were generated from both MRF and MAGiC, allowing for a direct comparison of the results. Further analysis revealed that for bone tissue, a significant and high positive correlation was observed for the T1 value generated by both MRF and MAGiC. However, the T2 value of bone tissue showed only a moderate positive correlation. Figure 3 displays the correlation plot for all tissues, providing a visual representation of the relationship between the T1 and T2 values of all tissues derived by MRF with that by MAGiC. The coefficient of correlations between the T1 and T2 values of all tissues was r = 0.83 (*p* < 0.0001) for T1 and r = 0.68 (*p* < 0.0001) for T2. After excluding the rectus abdominis and bladder wall tissues, a more positive correlation between MRF-derived T1 (r = 0.86) or T2 (r = 0.86) values with MAGiC-derived values was observed. These findings suggest that MRF and MAGiC are effective methods for measuring T1 and T2 values in various tissues, with strong correlations observed in most cases.

### 3.3. Quantitative Comparison between MRF-Derived T1 or T2 Value in Patients with and without Radiotherapy

The study conducted a comparison of T1 and T2 values of bone tissue between patients who had undergone radiotherapy and those who had not. Table 3 presents the mean T1 and T2 values of bone tissue generated from MRF for patients with and without radiotherapy. The results of the study indicated that there was a significant difference in the T1 values of bone tissue between patients with and without radiotherapy. Specifically, patients who had undergone radiotherapy had lower T1 values compared to those who had not (*p* < 0.001). This finding suggests that radiotherapy may have an impact on the T1 values of bone tissue. The study also found similar results for the T1 values of the ilium and femoral head tissue in patients with and without radiotherapy. Interestingly, there was a significantly lower T1 value in patients who had undergone radiotherapy compared to those who had not for both ilium (*p* < 0.001) and femoral head (*p* = 0.034) tissues.

### 3.4. Qualitative Comparison between MRF and Conventional MRI

We further compared the tissue contrasts generated from conventional MRI and synthetic MRF. The results showed that there were some agreements in the tissue contrasts for bone, muscle, and uterus in both T1-weighted and T2-weighted images. The tissue contrasts were visually better in the synthetic T2-weighted imaging than in the original T1 or T2 maps of MAGiC and MRF. This suggests that the synthetic MRF technique may provide more accurate and reliable tissue contrasts for detailed pelvic structures, including ilium, femoral head, gluteus, rectus abdominis, obturator, iliopsoas, erector spinae, uterus, cervix, bladder wall, cutaneous fat, for both MAGiC and MRF. The study also found that blood vessels showed overall high signal intensity in MRF-synthetic T2-weighted imaging, even in small vessels, such as superficial epigastric vessels. However, the agreements of T1 signal intensity were poor for fat and vessel, and the agreements of T2 signal intensity were fair to good for fat and poor for the vessel.

In comparison with the pubic symphysis, the signal intensity of fatty bone marrow showed T1-iso/hypo-intensity and T2-iso/hypo-intensity in both conventional MRI and MRF-synthetic weighted imaging. Similar signal intensity patterns were also noted when observing muscle and uterus. T1-hypointensity and T2-hyperintensity of urine were also noted. Excellent agreement between conventional MRI and tissue contrast generated from MRF was observed in bone, muscle, uterus, and urine for both T1W and T2W. The agreements of T1 signal intensity were poor for fat (κ = 0.152) and vessel; the agreements of T2 signal intensity were fair to good for fat (κ = 0.600) and poor for the vessel. Overall, the study demonstrated that the synthetic MRF technique might provide better tissue contrasts for detailed pelvic structures and that there were some agreements between conventional MRI and tissue contrast generated from MRF for bone, muscle, uterus, and urine. These findings may have important clinical implications, as accurate tissue contrasts are crucial for diagnosis and treatment planning in various pelvic pathologies.

## 4. Discussion

In the present study, we have demonstrated the effectiveness of MRF in simultaneously quantifying and visualizing tissue contrast on both T1-weighted and T2-weighted images in the female pelvis. This approach addresses the need for efficient T1 and T2 mapping in clinical applications, such as ovarian tumor characterization [25] and placental oxygenation detection in pregnant women [26]. MRF has also shown promise in examining pelvic organs, such as the uterine cervix [27], uterine body [28], rectum [29], urinary bladder [30], and even the femoral head [31]. In the uterine cervix, the T1 value measured from MRF is higher in cervical cancer than in normal cervical mucosa or stroma. The T2 value measured from MRF is lower in cervical cancer than in normal cervical mucosa [27]. Quantitative MRI allows T2 measurement in uterine tumors and can identify quantitative differences between uterine leiomyoma and uterine sarcoma [28]. Furthermore, quantitative MRI may help differentiate between the Funaki tissue types of uterine leiomyoma and may be useful in predicting treatment outcomes. Other potential applications in pelvic organs include evaluating prognostic factors of rectal cancer [29] and diagnosing overactive bladder [30], respectively. In the musculoskeletal field, T1 mapping of the femoral head is correlated with stress, which helps predict the fracture risk and indicates bone strength, while T2 mapping of the femoral head is related to re-fracture risk. Quantitative MRI adds value to the diagnosis of osteoporosis and the assessment of cancellous bone strength in the femoral head, besides standard bone mineral density [31]. Unlike previous studies that focused on small regions of interest in T1 or T2 maps, we examined the major target structures in the entire plane to eliminate sampling bias.

MRF and MAGiC are two different techniques used in medical imaging. MRF generates quantitative maps of tissue properties, while MAGiC acquires multiple images contrasts in a single scan. Our study found that there is a significant correlation between the T1 and T2 values generated by MRF and MAGiC in the pelvic tissue, in line with previous studies that showed good correlation in tissues of the abdomen, such as the liver and spleen [7]. However, there was an overall underestimate of T2 values in MRF compared to MAGiC scans, which is consistent with previous studies comparing MRF with conventional MRI [32]. The difference could be attributed to the fact that T2 values depend on B1, which can vary from day to day. Other factors that may contribute to the lower T2 values include the intravoxel dephasing [33], tissue microstructure, magnetization transfer [34], and slice profile [35].

MAGiC has a longer scan time compared to MRF, which can result in more artifacts from breathing motion, particularly in the pelvic organs of females. Additionally, MRF has demonstrated insensitivity to motion as the dictionary-matching process can reject such artifacts when a voxel is static for enough frames. Furthermore, MRF allows for the assessment of qualitative and quantitative data in a single sequence without misregistration. Our study validated MRF data using MAGiC and found significant correlations between T1 and T2 values generated from both techniques, especially for tissues other than the bladder and rectus abdominis. Drawing the thin bladder wall and rectus abdominis, along with partial volume effects at the border, may account for the error and difficulty in these areas. The application of MRF for imaging quantification of female pelvic structures shows promising potential, and diffusion imaging can also be obtained with MRF [36]. Additionally, MRF-synthetic T2WI provides bright visualization of blood vessels. Further research is necessary to explain this phenomenon, and this feature may aid in the easy identification of vessels.

The study demonstrated the effectiveness of MRF in simultaneously quantifying and visualizing tissue contrast on both T1-weighted and T2-weighted images in the female pelvis, addressing the need for efficient T1 and T2 mapping in clinical applications. MRF has shown promise in examining pelvic organs, such as the uterine cervix, uterine body, rectum, urinary bladder, and even the femoral head. Additionally, MRF allows for the assessment of qualitative and quantitative data in a single sequence without misregistration and insensitivity to motion artifacts. The study validated MRF data using MAGiC and found significant correlations between T1 and T2 values generated from both techniques, especially for tissues other than the bladder and rectus abdominis. The application of MRF for imaging quantification of female pelvic structures shows promising potential, and diffusion imaging can also be obtained with MRF.

Despite the promising potential of MRF in imaging quantification of female pelvic structures, there were several limitations in this study that should be acknowledged. Firstly, the sample size was small and heterogeneous, and no specific diseases were examined. This limitation may affect the generalizability of the study results to different patient populations with specific pelvic diseases. Larger sample sizes and studies focusing on specific diseases may be needed to further validate the findings of this study. Secondly, a phantom study was not conducted in this study, and references were used from external publications that also used MRF acquisition. Although external references were used to validate the MRF technique, it would have been more appropriate to have a phantom study to ensure the accuracy and consistency of the MRF technique [18,21]. Thirdly, there was no assessment of interobserver agreement to objectively evaluate the improved tissue contrast in synthetic MRI. The lack of interobserver agreement assessment could affect the reproducibility and reliability of the results. Additionally, the synthetic T2-weighted images produced by MRF provided a rough tissue contrast, which made it difficult to differentiate delicate structures in the female pelvis, such as the trilaminar mural stratification pattern of the uterus and cervix. Incomplete stimulations or flow-related artifacts may have affected the quality of the synthetic MRI. Therefore, further optimization of the MRF sampling strategy, as well as the use of more sophisticated signal models that account for the chemical shift, B0, or B1 correction, may be necessary for the pelvic region to improve the quality of the MRF synthetic T2-weighted images [37]. The absence of publicly available databases for MRF and conventional MRI in female pelvic tissue poses challenges and limitations in sample size, representation of diverse age groups and medical parameters, as well as the establishment of appropriate indications and optimal imaging protocols. Further research is necessary to overcome these limitations and enhance the clinical utility of MRF in female pelvic imaging.

## 5. Conclusions

In conclusion, the use of MRF allowed for the generation of T1 and T2 maps simultaneously, resulting in clear tissue contrast on both T1-weighted and T2-weighted images. The simultaneous generation of these maps using MRF provides a more precise diagnosis of diseases in the female pelvis. Furthermore, the potential of MRF in the field of female pelvis organ imaging is significant. The quantitative value and bone mineral density or muscle biological factors can be evaluated using MRF, which may aid in the detection of various conditions, such as tumors, fibroids, endometriosis, and pelvic inflammatory disease. MRF could be used as an opportunistic MRI in both patients with and without radiotherapy to evaluate the changes in the quantitative values and biological factors of pelvic organs. Overall, the findings of this study suggest that MRF is a promising imaging modality for the diagnosis of diseases in the female pelvis. Further research is needed to determine the full potential of MRF in this area and to develop standardized protocols for its clinical use.

## Figures and Tables

**Table 1:** Patient demographics and clinical characteristics.

Variable	Patients
Number	32 (100)
Age, median (y) ^a^	49 (26–83)
18–49	15 (47)
50~	17 (53)
Cancer	
Cervical	5 (16)
Endometrial	11 (34)
Ovarian	4 (13)
Leiomyoma	10 (31)
Others	2 (6)
Operative status	
Pre-operative	15 (47)
Post-operative	17 (53)
Radiotherapy	
With	5 (16)
Without	27 (84)

Numbers in parentheses are percentages. ^a^ Median (range).

**Table 2:** Mean T1 and T2 values of all tissue generated from MRF and MAGiC ^a^.

Tissue	T1 Value				T2 Value			
	MRF (ms)	MAGiC (ms)	r	*p* =	MRF (ms)	MAGiC (ms)	r	*p* =
Bone–Ilium	525.2 [± 188.4]	662.2 [± 145.8]	0.93	<0.001	79.13 [± 11.0]	108.5 [± 7.6]	0.63	<0.001
Bone–Femoral head	353.2 [± 74.9]	526.2 [± 67.0]	0.89	<0.001	90.3 [± 9.3]	130.7 [± 13.5]	0.39	0.027
Muscle–Gluteus	1359.0 [± 135.4]	1088.3 [± 64.9]	0.65	<0.001	41.7 [± 7.0]	50.3 [± 4.9]	0.68	<0.001
Muscle–Obturator	1563.9 [± 113.9]	1156.4 [± 45.5]	0.30	0.115	39.1 [± 8.1]	45.5 [± 3.8]	0.16	0.404
Muscle–Iliopsoas	1283.4 [± 194.7]	1467.0 [± 212.9]	0.13	0.491	49.0 [± 7.1]	47.4 [± 3.6]	0.02	0.931
Muscle–Erector spinae	1351.9 [± 252.2]	1227.4 [± 121.2]	0.22	0.243	56.9 [± 11.2]	56.6 [± 6.3]	0.27	0.150
Fat	313.5 [± 17.1]	492.8 [± 22.4]	<0.01	0.998	97.1 [± 10.8]	106.3 [± 9.1]	0.29	0.103
GYN–Uterus	1484.1 [± 119.0]	1522.1 [± 245.1]	0.74	0.003	62.2 [± 18.5]	77.1 [± 15.7]	0.76	0.001
GYN–Cervix	1455.9 [± 163.1]	1495.8 [± 217.5]	0.69	0.004	57.1 [± 15.6]	68.7 [± 11.4]	0.68	0.005

Numbers in parentheses are standard deviations. ^a^ Data are means. r values are correlation coefficients. *p* values are the significance of r. MRF = Magnetic resonance fingerprinting. MAGiC = Magnetic resonance image compilation. GYN = Gynecological.

**Table 3:** Mean T1 and T2 values of bone generated from MRF in patients with and without radiotherapy ^a^.

	Radiotherapy		*p* =
	With (ms)	Without (ms)	
Bone–Ilium			
T1	322.2 [± 16.0]	561.0 [± 185.2]	<0.001 *
T2	83.7 [± 5.2]	78.1 [± 11.8]	0.097
Bone–Femoral head			
T1	316.9 [± 12.0]	361.0 [± 80.7]	0.034 *
T2	87.7 [± 5.7]	90.3 [± 9.6]	0.251
Bone overall			
T1	319.5 [± 13.6]	461.0 [± 173.8]	<0.001 *
T2	85.7 [± 5.6]	84.2 [± 12.3]	0.352

Numbers in parentheses are standard deviations. ^a^ Data are means. MRF = Magnetic resonance fingerprinting. * *p* < 0.05.

## Data Availability

Data from this study will be available upon request.
